# Antitumor Anesthetic Strategy in the Perioperatory Period of the Oncological Patient: A Review

**DOI:** 10.3389/fmed.2022.799355

**Published:** 2022-02-18

**Authors:** Sonia Santander Ballestín, Andrea Lanuza Bardaji, Cristina Marco Continente, María José Luesma Bartolomé

**Affiliations:** ^1^Department of Pharmacology, Physiology and Legal and Forensic Medicine, Faculty of Medicine, University of Zaragoza, Zaragoza, Spain; ^2^Anesthesiology and Resuscitation, Clinic Hospital of Zaragoza, Zaragoza, Spain; ^3^General and Digestive Surgery, Hospital of Mérida, Mérida, Spain; ^4^Department of Human Anatomy and Histology, Faculty of Science, University of Zaragoza, Zaragoza, Spain

**Keywords:** anesthetic technique, oncologic surgery, tumor recurrence and metastasis, immunosuppression, inflammation, angiogenesis

## Abstract

The stress response triggered by the surgical aggression and the transient immunosuppression produced by anesthetic agents stimulate the inadvertent dispersion of neoplastic cells and, paradoxically, tumor progression during the perioperative period. Anesthetic agents and techniques, in relation to metastatic development, are investigated for their impact on long-term survival. Scientific evidence indicates that inhaled anesthetics and opioids benefit immunosuppression, cell proliferation, and angiogenesis, providing the ideal microenvironment for tumor progression. The likely benefit of reducing their use, or even replacing them as much as possible with anesthetic techniques that protect patients from the metastatic process, is still being investigated. The possibility of using “immunoprotective” or “antitumor” anesthetic techniques would represent a turning point in clinical practice. Through understanding of pharmacological mechanisms of anesthetics and their effects on tumor cells, new perioperative approaches emerge with the aim of halting and controlling metastatic development. Epidural anesthesia and propofol have been shown to maintain immune activity and reduce catecholaminergic and inflammatory responses, considering the protective techniques against tumor spread. The current data generate hypotheses about the influence of anesthesia on metastatic development, although prospective trials that determinate causality are necessary to make changes in clinical practice.

## Background

Cancer is one of the main causes of morbidity and mortality in the world. The number of patients diagnosed with tumor pathology grows exponentially year by year, has increased in incidence from 4 million cases worldwide in 2012 to 19.3 million in 2020. The population estimates point to as many as 29.5 million cancer cases worldwide in 2040 (1, 2).

Currently, surgical resection is considered the most effective method of removing the primary tumor (3) and to increase long-term survival in the vast majority of solid tumors (4). Even so, tumor recurrence and the development of metastases are very frequent even after surgical treatment, and are responsible for up to 90% of cancer-related mortality (5, 6).

Surgery (with or without chemotherapy and/or associated radiotherapy) is an increasingly common strategy used in the therapeutic management of cancer. It is known that during the perioperative period, surgery-induced stress responses and anesthetic-induced immunosuppression may play a critical role in the establishment and growth of metastatic lesions (7–10). During surgery, the tissue microenvironment is disrupted, and the perioperative period could create an environment that promotes survival, proliferation, and progression of residual cancer cells (11).

The hypothalamic–pituitary–adrenal (HPA) axis and sympathetic nervous system (SNS) regulate immune responses, and surgery-induced or anesthesia-induced activation of these two systems may facilitate metastasis through several tumor-derived soluble factors (12).

In recent years, accumulating evidence from preclinical studies suggests that adrenergic-inflammatory pathways may contribute to cancer progression. These possible pathways implicated could be modulated by adapting surgical and anesthetic technique (13–17).

Taking all these data as justification and as a basis, the objectives set out in this review address anesthetic agents and techniques used during oncological surgery to promote metastatic development and investigate whether “antitumor” techniques increase the survival of the oncological patient.

## Pathogenesis of Tumor Metastases

The development of metastasis is a process of dissemination of neoplastic cells from the primary tumor to other different organs, through the blood and/or lymphatic vessels (18, 19). This process depends on both the intrinsic properties of the tumor line and the host's immune response. During the perioperative period, tumor cell dispersion may be favored, even though complete macroscopic cytoreduction is achieved (19). Once the metastatic invasion cascade has started, a series of successive and interdependent steps lead to the activation of a metabolic cascade related to cell proliferation, growth, and apoptosis (3, 20).

Host immunity plays a very important role in lowering the rates of tumor survival. Natural killers (NKs) are primarily responsible for eliminating the neoplastic cells in the bloodstream (21). Both animal and human studies suggest that NK cell activity plays an essential role in disease-free survival after surgery (22). Regardless of factors, such as age, sex, differentiation, or tumor grade, the decreased NK cell activity predicts a high risk of tumor recurrence (23). Therefore, it should be noted that in cases of immunosuppression less activity and/or a number of NK cells, the host has a lower ability to cope with neoplastic cells, and consequently, the probability of metastasis increases (6).

The uncontrolled transformation and proliferation of neoplastic cells activate several mechanisms that promote the growth of new capillaries providing nutritional support to the tumor. The hypoxia-inducible factors (HIFs) promote the release of proangiogenic factors by the tumor, among which the vascular endothelial growth factor (VEGF) and the prostaglandin E_2_ (PGE_2_), and antiangiogenic factors decrease. Thus, the tumor cells will have the necessary support to reenter the systemic circulation, from where they will be transported to other organs and will start the cell proliferation cycle again from the beginning (24, 25).

The tumors release soluble factors into their microenvironments to block cell-mediated immunity (CMI) surveillance and facilitate tumor growth and metastasis (26). HPA axis and SNS activation by anesthetic agents suppress CMI and the release of catecholamines and PGE_2_. These factors increase immunosuppressive cytokines, soluble factors, and proinflammatory cytokines, which promote tumor angiogenesis and metastasis (27–29).

The importance in clinical practice of these data is that probably any process that promotes angiogenesis, neuroendocrine, and inflammatory responses, or immunosuppression, will favor the development and formation of premetastatic niches, and thus provide the necessary microenvironment for the training of new metastases (6, 23, 30). It is vital to avoid the activation of these molecular pathways, as well as therapies specifically directed against the establishment of premetastatic niches are potential candidates to be considered new treatments against metastatic development. Thus, the new lines of clinical research suggest new possibilities in the prevention of metastatic process and development (30).

## Anesthetic Drugs and Surgery: Molecular Tracks Involved in the Development of Metastases

The pharmacokinetic and pharmacodynamic knowledge of the anesthetic agents currently used has allowed us to know the differences between the effects of intravenous and inhaled drugs in terms of inflammation, immune system, and tumor development. Recent studies suggest that anesthetic drugs probably influence the metastatic process. These effects take on clinical importance by supposing a new route to counteract the dispersion and proliferation of residual tumor cells released during the surgical act (22).

It is known that the perioperative period (from a few days before and after surgery) represents, while treating a patient with cancer, a moment of maximum risk in the development of metastases (31). The possible magnitude of the impact of this perioperative vulnerability is reinforced by the fact that more than 60% of patients with cancer require surgical treatment, and more than 80% are exposed to anesthesia during the course of the disease. For this reason, we can consider the perioperative period as a new opportunity to eradicate and control residual tumor cells before they metastasize, with the goal of improving results both in terms of cancer survival and disease-free period, and the patient's life (32).

Surgical resection is the “gold standard” of treatment for most patients with solid tumors, often combined with other options, such as chemotherapy, radiotherapy, or biological therapies. However, even after resection with histological-free margins, there is a minimal clinically undetectable residual disease that conditions the dispersion of tumor cells during surgery (33). Based on these data, the appearance of cells with criteria for malignancy in peripheral blood within 24 h of oncological surgery was analyzed in a study, and it was observed that patients with blood-detectable tumor cells had a lower disease-free time (43.9 months) compared with patients who had no detectable cells (80.5 months). These results show how surgery promotes the release of neoplastic cells into the bloodstream (34), although it is true that new studies indicate that this effect decreases with the incorporation of new minimally invasive surgery techniques (33).

Surgery and anesthesia generate a stress response in the organism based on neuroendocrine stimulation of the HPA and SNS, through which it induces the release of cortisol and catecholamines (35). Prostaglandins and catecholamines can activate receptors, such as β2-adrenergic (36) and cyclo-oxygenase-2 receptors (37), that may have direct pro-tumor effects. Tissue trauma and inflammation lead to the release of cytokines (including interleukin-6 [IL-6] and PGE_2_) that also cause inhibition of NK cells.

*In vivo* studies show that tumor cells overexpress β-adrenergic receptors, and their binding to the released catecholamines activates a signaling circuit of cyclic Adenosine monophosphate (cAMP), and intracellular calcium increase that improves the transcription of premetastatic factors, such as HIF, VEGF, and matrix metalloproteinases (23). These mediators create a favorable microenvironment and have been shown to significantly increase tumor progression in breast, colon, ovarian, or prostate cancer models (32).

Through other molecular pathways, neuroendocrine factors released in response to stress inhibit other cellular mechanisms of cell repair. DNA promotes the continuity of mutations in neoplastic cells and alters apoptosis (12).

**Figure 1:**
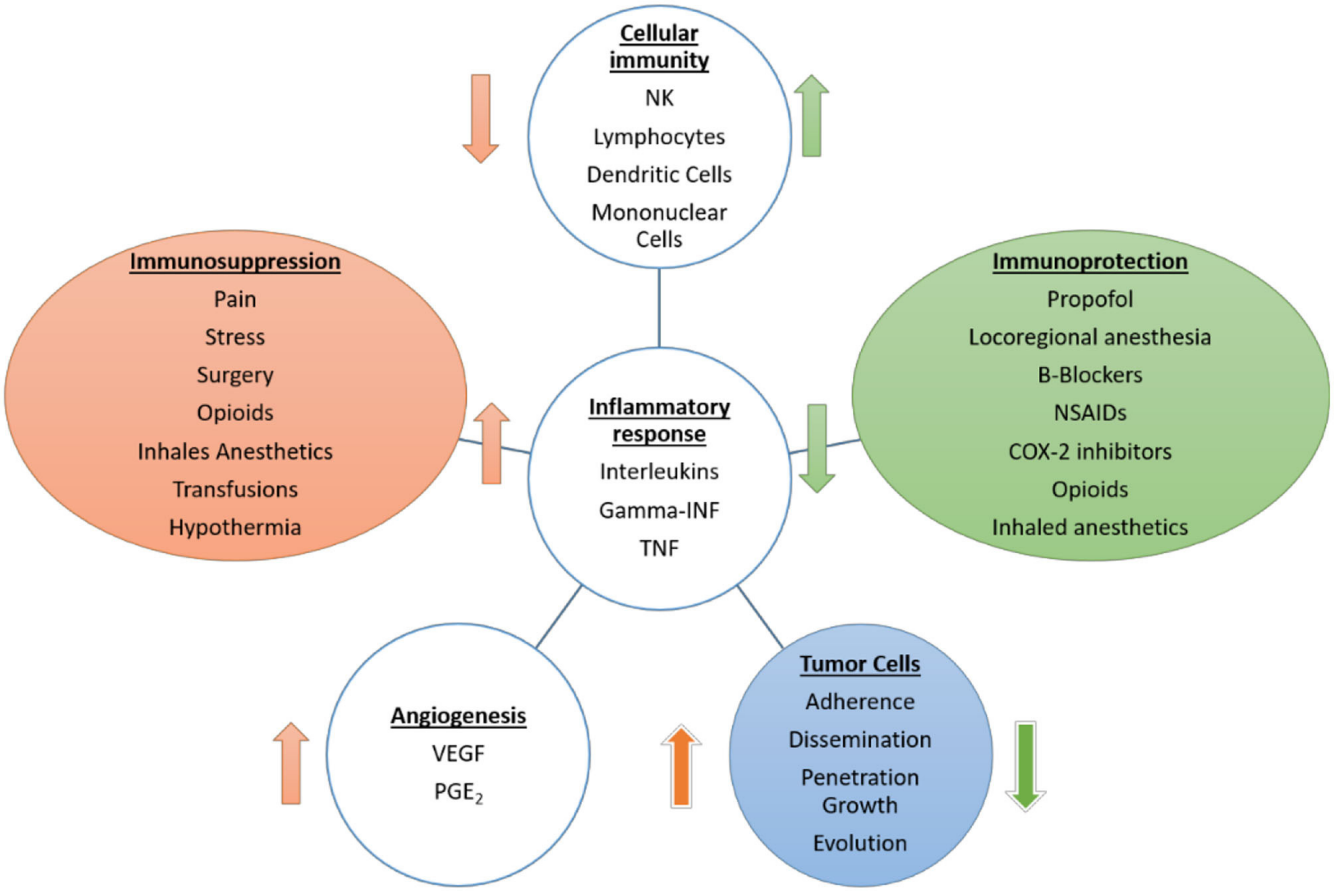
Effects of anesthetic drugs, surgery, and other perioperative factors on cellular immunity, inflammatory response, and angiogenesis as mechanisms involved in metastatic development. The scheme reflects the deleterious effect on the immune system and the increase in the inflammatory and proangiogenic response by factors, such as surgery, opioids, or inhaled anesthetics.

All of these mechanisms are summarized in Figure 1. The response to perioperative stress can have systemic effects and lead to the patient's vulnerability to tumor recurrence. However, there are many other perioperative factors to consider due to their known effect on the immune system and metastases, apart from surgery *per se*, such as hypothermia, blood transfusions, pain, stress, volatile anesthetics, or opioids. These aspects contribute toward the explanation of how the perioperative period of oncological surgery, paradoxically, can favor the development of distant metastases (38).

This high-risk perioperative time window can be a critical period where a multidisciplinary team of surgeons, anesthesiologists, and oncologists can be actively involved by choosing an “antitumor” perioperative technique, and thus help to improve oncological survival for long-term patients (39).

## Materials and Methods

This review has been carried out to summarize the available results of experimental studies. To identify the eligible articles, we performed a systematic literature search on the PubMED database.

We used the following search terms: “anesthesia and cancer recurrence and metastasis” OR “aesthetic mechanism, and cancer and surgery” OR “cancer and metastasis and physiopathology and development and mechanism” OR “perioperative period and cancer and metastasis and development” OR “surgery and anesthesia and immunosuppression and metastasis” OR “intravenous anesthetics and metastasis and perioperative” OR “volatile anesthetics and metastasis and perioperative” OR “opioids and metastasis and perioperative” OR “propofol and metastasis and perioperative” OR “regional anesthesia and metastasis and perioperative” OR “local anesthetics and metastasis and perioperative” OR “anesthetic and analgesic techniques and metastasis” OR “beta blockers and COX inhibitors and perioperative”; carrying out different combinations of them to find the articles included and epidemiological studies. The selected search criteria were as follows: full text, 5 years, clinical trial, meta-analysis, review, and humans.

The publication criteria extended to 10 years for the search “Anesthetic technique and metastasis and pathogenesis,” because the studies published during this period established the pathophysiological basis considered in this review. This information is summarized in Table 1. The relevant references identified in the selected articles were also reviewed.

**Table 1:** Results obtained for each search term and criteria.

**Searching criteria**	**Searching terms**	**Plaform**	**Results**
Full text 5 year's clinical trial meta-analysis review humans	Anesthesia and cancer recurrence and metastasis	PubMed	91
	Anaesthesic mechanism and cancer surgery	PubMed	22
	Cancer and metastasis and physiopathology and development and mechanism	PubMed	34
	Perioperative period and cáncer and metastasis and development		43
	Surgery and anesthesia and immunosupression and metastasis	PubMed	7
	Intravenous anaesthesics and metastasis and perioperative	PubMed	9
	Volatile anaesthesics and metastasis and perioperative	PubMed	7
	Opiods and metastasis and perioperative	PubMed	19
	Propofol and metastasis and perioperative	PubMed	9
	Regional anesthesia and metastasis and perioperative	PubMed	16
	Local anaesthesics and metastasis and perioperative	PubMed	15
	Anaesthesic and analgesic techniques and metastasis	PubMed	23
	Beta blockers and COX inhibitors and perioperative	PubMed	3
Full text 10 year's clinical trial meta-analysis review humans	Anaesthesic and techinique and mechanisms and pathogenesis	PubMed	21

## Results

### How Do Anesthetic Drugs Influence the Metastasic Process?

To block the tumor spread and growth during the perioperative period, numerous retrospective studies investigated the molecular bases involved in this process and provided essential information on the mechanisms of interaction between anesthetics and the tumor cycle. The results obtained could, in turn, be the subject of new prospective studies that determine a significant causal relationship. This new link established between the anesthetic technique and the development of metastasis lays the foundation for a series of “antitumor” anesthetic proposals that allow the anesthesiologist to participate in the optimization of oncological results (recurrence rate and metastasis) (39). Therefore, the mechanisms underlying cancer recurrence after surgery are not completely understood. Cancer cells exist in a complex tissue microenvironment involving an interplay of surrounding noncancerous stromal cells, extracellular matrix, immune system cells, chemokines, and cytokines (40).

#### General Anesthetics

Depending on their route of administration, general anesthetics are divided into volatile (nitrous oxide, halogenated) or intravenous (propofol, ketamine, benzodiazepines). It is known that at the cellular level, general anesthetics exert their effects on the central nervous system by hyperpolarizing the neuronal membranes, thus decreasing their activity and responsiveness and altering synaptic transmission. The channels through which hyperpolarization occurs vary between different anesthetic agents, giving them molecular and cellular selectivity, and differences of pharmacological profiles (41).

##### Inhaled or Volatile Anesthetics

Inhaled anesthetics when inhaled through the respiratory system produce general anesthesia (41). Nowadays, halogenated agents, such as sevoflurane, isoflurane, or desflurane, are the most widely used in inhaled or combined general anesthesia (42). In routine clinical practice, its main use lies in the maintenance of anesthesia, with intravenous anesthetics being preferred in induction (41).

The volatile anesthesia has been shown to have effects on the immune system and the inflammatory response that may directly affect cancer cell survival (43–45). Regarding their impact on metastatic development, these agents participate in the formation of a proinflammatory microenvironment, increasing cytokines (as IL-1, IL-8) (46), modulating cellular targets on immune cells (such as neutrophils, macrophages, and NK cells) (47) and upregulating anti-apoptotic pathway signaling (48). The mediators implied favor the interaction between reactive oxygen and nitrogen species and cellular DNA, causing genomic damage and acquisition of mutations in cells. Furthermore, the inflammatory response favors angiogenesis, cell proliferation, and initial tumor growth, providing the perfect environment for tumor development (42).

Another important molecular pathway in this process, on which numerous studies have focused (49–51), is the activation of the -HIF 1α. Its relevance lies in the fact that the low oxygen concentrations to which the tumor cells are subjected represent the main stimulus to induce their expression, and thus a cascade of molecular mechanisms that guide toward proliferation, cell migration, angiogenesis, and hematogenous spread.

Expressing HIF-1α in the center of the tumor mass activates an adaptive response to hypoxia that decreases apoptosis and increases tumor cell survival, which in turn induces resistance to certain oncological treatments. Therefore, if this molecular pathway is activated as little as possible, or even blocked, it could act directly on metastatic development (49).

By combining the action of inhaled anesthetics on all these molecular pathways, the end result is the creation of a premetastatic niche in which the progression, proliferation, migration, and invasion of tumor cells are stimulated (50). When analyzing these mechanisms, several recent retrospective studies found a significant reduction in survival when patients received inhaled anesthesia, being this more marked in some specific tumor types (51).

##### Intravenous Anesthetics

The term “intravenous anesthetics” includes substances with anesthetic properties that are not gases and that are administered intravenously (41). Intravenous anesthesia was used in 1930, with the introduction of barbiturates, and these drugs are currently considered essential in deep sedation and general anesthesia, whether balanced with the use of inhaled agents, or total intravenous anesthesia (46).

**Figure 2:**
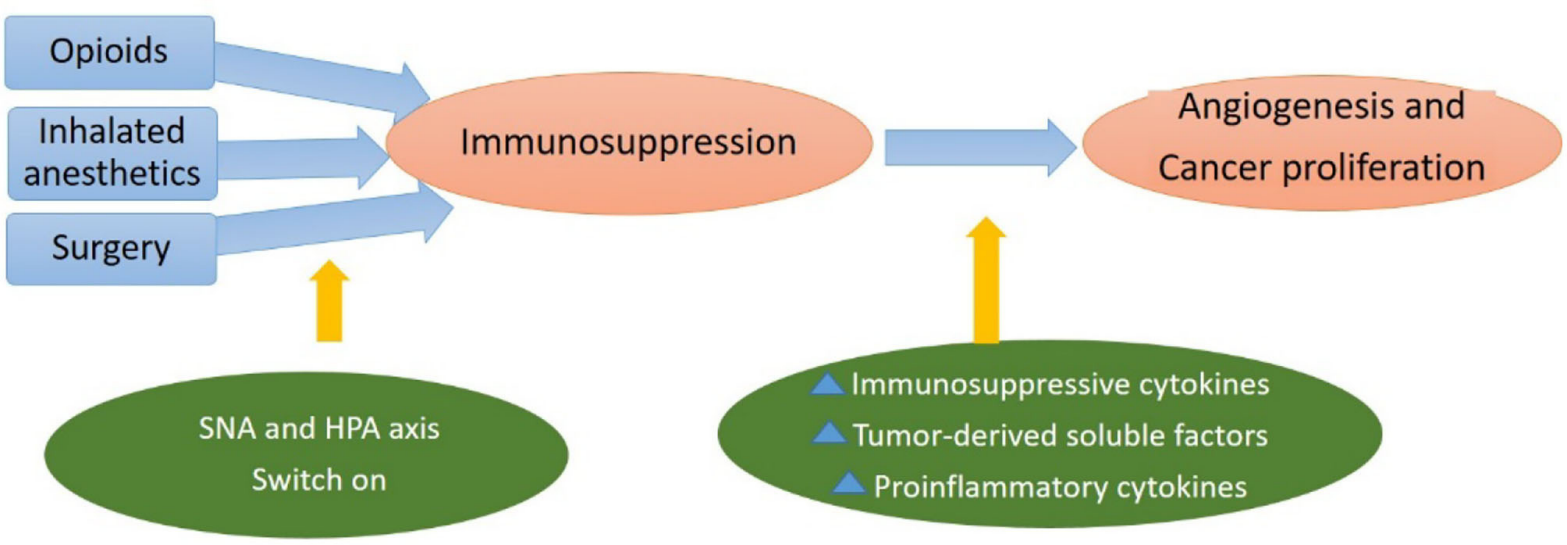
Hypothalamic–pituitary–adrenal (HPA)-axis and sympathetic nervous system (SNS) activation by anesthetic agents suppress cell-mediated immunity and release factors increasing immunosuppressive cytokines, soluble factors, and proinflammatory cytokines, which promote tumor angiogenesis and metastasis.

• Propofol: This is a short-term intravenous anesthetic agent, which has particular properties; thanks to its lack of chemical relationship with the rest of intravenous agents. This agent is widely used in anesthesia for all types of surgeries, both for induction and maintenance since it causes an anesthetic effect with loss of consciousness. The anesthetic effect depends on the activation of the receptors in the gamma-aminobutyric acid (GABAa) located mainly in the central nervous system. The interaction of propofol with its binding site at the GABAa receptor supposes the opening of the channel and the entrance of chlorine to the cell (hyperpolarization) (41).
  The gamma-aminobutyric acid receptors predominate the central nervous system level, but can also be found in other peripheral organs and various types of tumor cells, where the gabaergic signal controls the proliferation, differentiation, and migration of cells, including tumor cells (52).
  When investigating the link between this anesthetic and the inflammatory response, significant results were obtained; thanks to their inhibitory effect on cyclooxygenase 2 (COX-2). There is less production of PGE_2_ by human monocytes *in vitro*, (53) and it decreases the concentration of other proinflammatory cytokines, such as IL-10, IL-6, or tumor necrosis factor α (TNF-α) (46). Due to all the mentioned mechanisms, propofol generates a lower inflammatory response when compared to inhaled agents, which could improve the oncological prognosis of patients (53).
  In contrast to other intravenous anesthetics, propofol does not decrease NK cell activity in rodents (53). Neither does it decreases lymphocytes, neutrophils, or phagocytes in healthy volunteers; which is what allows the immune system to maintain the total capacity of action (46).
  Numerous studies evaluate the effects of propofol on the immune system, and the most recent research indicates that the basis of propofol's antineoplastic effect lies both in its absence of an immunosuppressive effect and in the lower magnitude of the triggered inflammatory response when compared with volatile anesthetics (32, 46). The correct use of propofol in oncological surgery allows the reduction of the necessary dose of inhaled anesthetics. Thus, the use of propofol is considered as an “antitumor” or “immunoprotective” technique. Regarding this hypothesis, a retrospective long-term study investigated how mortality after surgical treatment in solid tumors varied depending on the type of anesthetic used, with the mortality rate being 50% higher in the group of inhaled agents with respect to propofol, and thus demonstrating a clear relationship between anesthetic technique and patient survival after cancer surgery (54). While new prospective clinical studies are being conducted, the currently available evidence points that propofol-based anesthesia is the preferred anesthetic technique in oncological surgery (32).
• Opioids: Opioid analgesics are a family of substances characterized by a selective affinity for opioid receptors, mainly located in the central nervous system and digestive system. Its activation produces different effects, such as sedation, analgesia, and respiratory depression (17). Opioid analgesics are reported to inhibit cellular and humoral immune function and increase angiogenesis (55). Opioid analgesics are potent pain-relieving agents routinely used for pain management in patients with cancer, inducing their analgesic effect; thanks to a high affinity for μ receptor at the central nervous system level. However, the μ receptor is also expressed in nonneuronal tissues (including endothelial, immune, and tumor cells). High μ receptor expression level has been observed in human tissues with lung, prostate, and colon cancer (56). When overexpressed in tumor cells and the opioid ligand binds, the neuroendocrine and inflammatory cascades are activated (stress response), and catecholamine synthesis is increased, nitric oxide (NO) and PGE_2_. The secretion of all these factors at the systemic level is related to increased angiogenic activity and metastatic progression (17).
  The direct effects on immune function may occur *via* opioid receptors, such as the μ receptor, or nonopioid receptors expressed by immune cells, including NK cells (57). In retrospective studies, l-opioid receptor overexpression is associated with worse outcomes in patients with prostate cancer and esophageal squamous cell cancer (58, 59).
  The effects of opioids at the immune level are added to the neuroendocrine stress response to lead to the imbalance of the system toward immunosuppression, as we can see in Figure 2 (17). Morphine is the opioid with the highest affinity for the μ3 receptor, which is the main cause of modulating cellular and humoral responses through the decrease in the activity of macrophages and NK cells. For this reason, morphine causes a greater deleterious effect on the immune system (60). Compared to morphine, synthetic opioids, such as fentanyl or remifentanil, have been shown to induce immunosuppression to a lesser degree, because their binding to the μ3 receptor is significantly less. In clinical practice, fentanyl is the opioid that has shown the least effect on NK cells and cytotoxic T lymphocytes (61).
  In contrast to the results that associate morphine with cancer progression, Doornebal et al. showed in metastatic invasive lobular and HER2+ breast cancer mice models that doses of morphine used as analgesic did not affect mammary tumor growth, angiogenesis, and composition of immune cells in the presence or absence of surgery-induced tissue damage (62).

#### Local Anesthetics

The local anesthetics block nerve conduction in a reversible way. These agents produce both use- and voltage-dependent inhibition of voltage-gated sodium channels (VGSCs), blocking channel resting, open, and inactivated states (where inactivated states are thought to bind with the highest affinity). There are two main classes: amides and esters. Amide types, in particular lidocaine, have more systemic anti-inflammatory benefits and effects on immune cells compared with other local anesthetics (63). Inflammatory mechanisms may play an important role in the development and growth of cancer metastases (64).

The voltage-dependent sodium channels have also been found in neoplastic cell membranes, where their effect is correlated with invasion and metastasis formation. When those are blocked by local anesthetics, it is possible to inhibit the growth, invasion, and migration of tumor cells (65). In addition, local anesthetics increase intracellular calcium concentrations, producing free radicals and causing structural and functional damage to mitochondria and cell membranes, resulting in tumor cell apoptosis (66).

The local anesthetics inhibit the afferent drive that carries sensory information regarding pain, lowering cortisol and catecholamine levels after surgery (65). Through this blocking of pain, it is possible to attenuate, or even prevent, many of the adverse effects caused by the neuroendocrine response to surgical stress (67).

The use of local anesthetics might potentially reduce the risk of immunosuppression and provide selective inhibitory effects on cancer cells, often *via* methods distinct from sodium channel blockade (68). Several studies have shown molecular evidence related to tumorigenesis, which might be a possible explanation of the beneficial effects in the use of local anesthetics:

- Effects on cell division: Epidermal Growth Factor–Associated Effects: Epidermal growth factor receptor (EGFR) is a tyrosine kinase receptor that regulates cellular proliferation and differentiation of epithelial cells and tumors, including head and neck, breast, colorectal, and lung cáncer (69). Chang et al. (70) found that local anesthetics preferentially induced the EGFR pathway in breast cancer cells compared with nontumorigenic epithelial cells *via* caspase-dependent extrinsic and intrinsic apoptosis. These cells have a greater expression of EGFR compared with epithelial cells. Thus, activation of the EGFR leads to the increased downstream activity of caspases 8 and 9, which leads to apoptosis of breast cancer cells (70).
- Inhibition of voltage-gated sodium channels: These channels are uniquely expressed in active breast, colon, and prostate cancers (71–73). Local anesthetics inhibit voltage-gated sodium channels preferentially in the inactivated state, leading to reduced cellular activity, including invasion and cellular motility (71).
- Mitochondrial inhibition: Mitochondrial metabolism plays an essential role in tumor growth, survival, and spread *via* changes in ATP levels (74). Some studies have shown that local anesthetics inhibit different mitochondrial complexes in gastric cancer, which leads to decreased ATP. The ATP levels correlate well with gastric cancer growth and survival (75). Chang et al. (70) found that lidocaine and bupivacaine affect mitochondria, leading to reduced cell growth and colony formation in high concentrations of thyroid cancer cells. Local anesthetics increase proapoptotic Bax expression and reduce Bcl-2 expression, leading to altered ratios of pro and antiapoptotic proteins, in particular, cytochrome C (76).
- Calcium influx: Calcium is a critical regulator of cell migration, in particular tumor cells from breast, prostate, and ovarian cancer. Calcium signaling controls cancer cell progression and apoptosis *via* the transient receptor potential subfamily V member 6 (TRVP6) channel. Jiang et al. (77) showed that lidocaine reduced TRPV6 expression by 50–80% in breast cancer cells, showing a decreased cell viability, reduced cell migration, and cell division in a concentration-dependent manner.
- TNF-α: TNF-α increases the expression of intracellular adhesion molecule−1 (ICAM-1), a receptor required for leukocyte adhesion and tumor invasion. ICAM-1 assists with tumor extravasation in lung cancer *via* the binding of neutrophils. TNF-α also activates Src protein tyrosine kinase, a regulator of endothelial permeability, which is involved in the extravasation of cancerous cells, promoting angiogenesis, proliferation, and invasion of cancer cells. Piegeler et al. (78) demonstrated that both ropivacaine and lidocaine coadministered with TNF-α significantly decreased Src activation and ICAM-1 phosphorylation, showing the antimetastatic potential of both local anesthetics.
- Cell Cycle–Dependent Effects: Le Gac et al. (79) evaluated the impact of ropivacaine on human hepatocellular carcinoma (HCC) cells. Ropivacaine halted progression at the G2 phase of the cell cycle (74, 79). Zhang et al. (80) found that in lung cancer treated with lidocaine, there is reduced cell proliferation *via* Golgi transport 1a (GOLT1A). GOLT1A was significantly elevated in patients with lung adenocarcinoma and was related to poor prognosis and adverse pathological stage (80). Downregulation of GOLT1A reduced proliferation and induced cell cycle arrest.
- Migration: Local anesthetics affect the migration of cancerous cells. Low concentrations of bupivacaine (10–50 mM) reduced migration of gastric cancer cells *via* the Ras homolog gene family member A (RhoA) and myosin light chain (MLC) pathways (81). A low level of migration can limit the effects of metastatic disease.
- Demethylation Effects: Villar-Garea et al. showed that binding of procaine causes a hypomethylation and demethylation of tumor suppressor genes with hypermethylated CpG islands in breast cancer cells (82). They showed that the occurrence of demethylation was simultaneous with the reduction in the growth of breast cancer cells. Similar findings were also shown in leukemia (83) and HCC (84). Other studies by Lirk et al. (85) found smaller changes in some types of breast cancer cells, showing that less methylation in cells may lead to a less significant role of local anesthetics.

It is worth highlighting that *in vivo* studies carried out in mice were about lidocaine inhibited migration of breast cancer cells compared with normal breast epithelial cells (86). Lidocaine has also been shown to reduce peritoneal carcinomatosis (86) or pulmonary metastasis in mice with colon cancer (87).

A study of lidocaine administration in women with cervical cancer undergoing radical hysterectomy showed that the ratio of interferon-c to IL-4 was preserved in these women when compared with those who did not receive it (88). Thus, there was a protective effect in patients receiving lidocaine infusions undergoing radical hysterectomy, which may be protective against the recurrence of metastasis. Further, there was also an inhibition of high mobility group box-1 protein (HMGB1) production in these women, which could be another helping mechanism to reduce the metastasis (89). Thus, there is evidence that implicates the use of local anesthetics in reducing cancer recurrence *via* many different mechanisms. Tumor progression may be indirectly affected by local anesthetics through diminishing neuroendocrine response to surgery, preserving immunocompetence, and reducing the use of opioids as an antitumor strategy in the perioperatory period (90).

#### General and Regional Anesthesia

Nowadays, combining general and regional anesthesia in major surgery is a usual clinical practice. Numerous retrospective studies and randomized trials indicate potential perioperative benefits when introducing regional anesthesia in oncological surgery. This combined technique allows the reduction of the requirements of inhaled anesthetics and opioids, indirectly reducing the immunosuppression that their use entails (66, 91).

Regional anesthesia has been shown to suppress the surgical stress response (92). During the perioperative period, attenuation of the stress response may reduce immunosuppression. It could minimize the use of volatile anesthesia and opioid requirement due to the improved pain control and therefore preserves the immune system's capacity to eliminate residual cancer cells (93).

Epidural anesthesia has been associated in multiple studies with an improvement in all-cause survival after cancer surgery. However, the results regarding the tumor recurrence rate are contradictory (40). A meta-analysis found a positive association for neuraxial anesthesia and improved survival compared with general anesthesia (Hazard Ratio (HR) 0.85, 95% CI: 0.741–0.981, *p* = 0.026), particularly in colorectal cancer surgery (HR 0.65, 95% CI: 0.430–0.991, *p* = 0.045), as well as increased recurrence-free survival (HR 0.846, 95% CI: 0.718–0.998, *p* = 0.47) (33). These results are explained because epidural anesthesia allows to preserve a greater number and activity of NK cells during the perioperative period, offering certain antitumor properties in its clinical application in oncological surgery (65). Randomized controlled trials comparing regional techniques with general anesthesia in patients with cancer reported a significant reduction in NK-cell and T-cell activity compared with patients who received general anesthesia alone (94, 95).

Thus, regional anesthesia could be considered as a potential technique to reduce surgical stress response, improve pain control, and reduce postoperative complications (67), proposing oncological benefits.

The decision on whether to use epidural anesthesia in combination with general anesthesia must be taken into account on many occasions. Even if the results of prospective randomized studies demonstrate a reduction in tumor recurrence when using local anesthetics in locoregional techniques, these results may not be universally applicable, as they are a challenge to implement new specific strategies in a variety of patients and tumors (33). Therefore, even having found a solid theoretical basis, molecular mechanisms for these effects are incompletely understood, the real benefits in clinical practice have not been definitively demonstrated, and the choice to use regional anesthesia should be relegated to other reasons until we have data with more clinical evidence (67).

**Figure 3:**
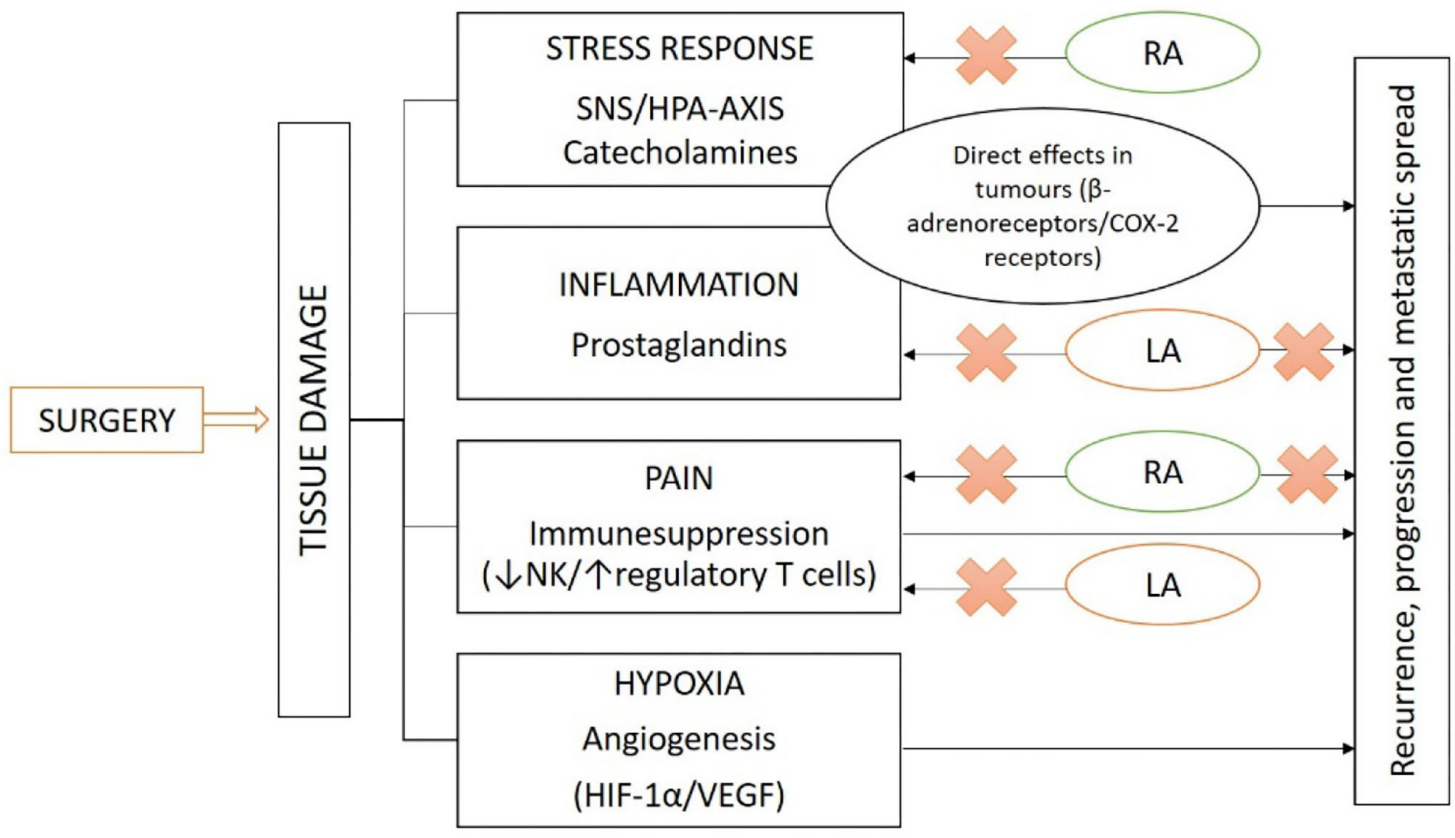
Oncological surgery activates tissue damage, creating an environment of physiological stress response, inflammation, pain, and hypoxia. These cause the release of hormonal mediators (catecholamines, prostaglandins, and growth factors), activating receptors with direct pro-tumor effects and contributing to immune suppression. RA and LA block pain, attenuating many of the adverse effects caused by the neuroendocrine response to surgical stress, proposing oncological benefits (RA, regional anesthesia; LA, local anesthesia).

Figure 3 shows a summary of factors contributing to immunosuppression, which may have direct pro-tumor effects and how local and regional anesthetics could be considered as potential techniques to reduce surgical stress, proposing oncological benefits.

### Impact of Anesthetic Drugs According to the Type of Cancer

In the development of this section, the most frequently diagnosed tumors in the world during 2018 have been considered according to the Spanish Society of Medical Oncology (SEOM) and the Spanish Network of Cancer Registries (REDECAN); highlighting the importance of lung, breast, colorectal, and prostate cancer nowadays. When reviewing the latest scientific evidence, it was considered relevant to include esophageal cancer because the results obtained were statistically significant and could have implications for future lines of research (1, 2). The results obtained depending on the different tumors are summarized in Table 2.

**Table 2:** Relationship studies between surgical procedure as cancer treatment, anesthetic technique adopted, and results.

**Surgical procedure**	**Type of tumor**	**Anesthetic technique**	**Authors**	**Patients**	**Year**	**Type of study**	**Oncological results**
Surgery with curative intention (colectomy)	Colorectal cancer	Propofol-based anesthesia vs. inhaled anesthetics	Lifang et al.	457	2013	Cohort study	Decreased tumor invasion capacity in rectal cancer, but not in colon cancer, with the use of propofol (50).
		General anesthesia + Epidural anesthesia vs. General anesthesia	Gupta et al.	655	2011	Cohort study	Reduction of all causes of mortality in the rectal cancer and Epidural anesthesia group, but not in the colon group (96).
		General anesthesia + Epidural anesthesia vs. General anesthesia	Xuan et al. Cummings et al.	132 42151	2014 2012	Cohort study Cohort study	Surgical treatment in patients older than 66 years: – Longer median survival with Epidural anesthesia (97). The recurrence rate does not vary between both groups. – Improved overall survival at 5 years in epidural group, without difference in cancer recurrence (65).
Mastectomy	Breast adenocarcinoma	General anesthesia + paravertebral block vs. Inhaled anesthetics + opioids	Exadaktylos et al.	129	2006	Retrospective study	Increased recurrence free survival in locorregional group at 3 years (88 vs. 77%) (98).
		Propofol-based anesthesia vs. Inhaled anesthetics + opioids	Hiller et al.	256	2017	Cohort study	Longer median survival in the propofol-based anesthesia group (32).
Resection surgery	Lung cancer	General anesthesia + Epidural anesthesia vs. General anesthesia	Xuan et al.	132	2014	Cohort study	EA is associated with decreased inflammatory response and endothelial permeability. Less dispersion of tumor cells (97).
		General anesthesia + paravertebral block vs. General anesthesia with opioids	Lee et al.	1729	2017	Retrospective cohort study	Paravertebral block was associated with be‘er overall survival, without difference in recurrence between the two groups (99)
Radical prostatectomy	Prostatic adenocarcinoma	General anesthesia + Epidural anesthesia vs. General anesthesia + opioids	Fodale et al. Behrenbruch et al. Scavonetto et al.	99 90 3284	2014 2018 2014	Randomized controlled trial. Cohort study. Retrospective matched cohort study	– 60% reduction in risk of tumor recurrence in the General anesthesia + Epidural anesthesia group (61). – Increased tumor progression and all-cause mortality higher in the General anesthesia group (91). – General anesthesia as unique technique is associated with increased cancer progression and mortality (100).
		Epidural anesthesia vs. General anesthesia	Gupta et al.	655	2011	Cohort study	Decrease in the rate of tumor recurrences (Valued as an increase in PSA) (96).
Esophagectomy	Esophageal cancer	Propofol-based anesthesia vs. inhaled anesthetics	Jun et al.	922	2017	Cohort study	Increased survival rate in the propofol group (101).
		General anesthesia + Epidural anesthesia vs. General anesthesia + opioids	Hiller et al.	140	2014	Survival study	Epidural was associated with better overall survival and recurrence 2 years free-survival (102).

#### Colorectal Cancer

Colorectal cancer surgery is one of the most comparative studies between anesthetics, evaluating the results in terms of survival and cancer recurrence. Many of the investigations propose the perioperative period as a unique window of opportunity to deal with immunosuppression induced by surgery (91, 103). In this context, propofol could have an antitumor effect on rectal cancer, since it reduces the invasion capacity of this type of neoplastic cells when it reaches clinically relevant concentrations, unlike in colon cancer (50).

Gupta et al. have studied a database of 655 patients and found a reduction of all causes of mortality in the rectal cancer and epidural anesthesia group, but not in the colon group, so they hypothesized that the protective effect of epidural anesthesia was associated with different types of tumor and location (96). Cummings et al. have recently carried out a prospective study on a database of 42,000 patients older than 66 years diagnosed with colorectal cancer. Through a multivariate analysis, they obtained a significant association between the use of epidurals and a higher mean survival [Odds Ratio (OR) 0.91 with 95% CI (0.87–0.94) and *p* < 0.001]. The recurrence rate did not vary between the two groups (65).

#### Breast Cancer

General anesthesia combined with epidural is increasingly used in clinical practice and has been shown to significantly reduce short-term recurrence and metastasis development in breast cancer, as well as improve oncologic survival (63). This is largely due to the fact that it is the anesthetic option that least stimulates the expression of HIF-1α, whose blood level after surgery is proportionally related to the development of short-term breast cancer metastases (50).

The association between the risk of tumor recurrence during the first 5 years after a mastectomy and the anesthetic approach used has been investigated, obtaining interesting results in several studies: the risk of recurrence decreases significantly when using propofol (32), as well as when using epidural anesthesia (104). Furthermore, in a recent retrospective study, propofol-based anesthesia during mastectomy is associated with improved survival compared with inhaled anesthesia (HR: 0.55, 95% CI: 0.31–0.97) (32).

#### Lung Cancer

In animal models, nitrous oxide (46), thiopental, and especially ketamine, have stimulated the development of metastases in lung carcinomas (50). However, there is no evidence of this effect when using sevoflurane, perhaps because it is the only inhaled agent that inhibits HIF-1α instead of stimulating it (3). By combining general anesthesia with epidural anesthesia, the decrease in inflammatory response and endothelial permeability was demonstrated, acting as a protective barrier against cell dispersion in lung carcinoma (65).

#### Prostate Cancer

The results obtained in the studies evaluating the anesthetic technique in patients with prostate cancer are conflicting. One study obtained significant results when studying the use of general anesthesia combined with epidural anesthesia or general anesthesia combined with opioids in radical prostatectomy (indicated for adenocarcinoma), showing a 60% reduction in the risk of tumor recurrence in the general anesthesia and epidural anesthesia group (64), and an increase in tumor progression and all-cause mortality in the general anesthesia group (39).

The use of epidural anesthesia has also been associated with the maintenance of normal prostate-specific antigen (PSA), whose positivity after a radical prostatectomy would be indicative of risk of recurrence or metastasis (96). However, other studies that have assessed long-term survival using epidural anesthesia have not obtained significant results (104, 105).

#### Esophageal Cancer

Jun et al. (101), in a retrospective observational study, compare the use of propofol and inhaled agents in esophageal cancer surgery, obtaining significant results in terms of improving the postoperative survival rate when using propofol. This is especially important because this type of cancer is frequently aggressive and has high rates of postoperative recurrence despite therapeutic improvements, which would considerably affect its forecast. The researchers attribute the significant results in esophageal cancer to the longer time it takes to perform esophagectomy, subjecting the patient to anesthetic drugs for a longer period of time compared with other surgeries (101). However, we should not ignore the need for new randomized studies that can establish a causal relationship.

### Potential Perioperatory Antitumor Interventions

The surgical-anesthetic stress response is mediated by catecholamines and prostaglandins during the perioperative period, and has been correlated with the increased risk of tumor recurrence and metastasis development. The identification of these factors as intermediaries in the metastatic process is the basis on which to initiate new studies that evaluate if their consideration in clinical practice is effective and recommended (106). Following this line of research, a recent international consensus on onco anesthesia marked the investigation of those perioperative factors that have shown a potential influence on cancer recurrence, with the main objective of incorporating new preventive measures into routine practice (32).

Animal studies and retrospective clinical investigations suggest blocking the tumor development pathways that are affected in the perioperative period, especially the inflammatory or neuroendocrine response, as an efficient therapeutic measure to improve oncological survival (106).

#### Locoregional Anesthesia and Propofol

One way to achieve sympathetic blockade during cancer surgery is neuraxial anesthesia. Its reducing effects on circulating catecholaminergic and inflammatory levels, and on perioperative immunosuppression, make it an “antitumor” anesthetic technique. The recent animal studies indicate that epidural anesthesia may decrease perioperative lymphatic flow and thus hinder the spread of residual cancer cells. The latest meta-analyses show how effectively perioperative neuraxial anesthesia, as long as it can be indicated, is associated with a survival benefit in patients with cancer, reducing the risk of tumor progression and mortality by 15% (HR 0.85, 95% CI: 0.75–0.94) (32).

These results conclude that both drugs and anesthetic techniques directly influence the immune system through molecular cascades involved in transient immunosuppression and the development of metastases. Compared with general anesthesia (inhaled anesthetics and opioids), locoregional techniques and propofol-based anesthesia have been shown to decrease surgical stress, immunosuppression, and angiogenesis. Even so, the complexity of all the metabolic pathways involved in this new link requires randomized clinical trials to obtain more data to establish a meaningful relationship, on which to base a future antitumor strategy on the anesthetic management of the oncological patient (3, 65, 66, 107).

#### Decrease in Opioid Use

In the early 2000s, morphine was shown to stimulate angiogenesis *in vitro*, being related to increased tumor growth *in vivo* in subsequent studies. In 2006, the first clinical study emerged on the possible effect of morphine on metastatic development after surgery. However, the available scientific evidence is still limited to retrospective studies, needing new randomized clinical studies that prospectively investigate different lines of action to avoid the unwanted effects of the opioid drugs (108).

This relationship becomes critically important when we analyze the clinical management of the patient with cancer, since the use of opioids is often resorted to (both in the anesthetic field and in the pain treatment). The importance of this possible relationship may lie in the fact that one of the most frequent symptoms in the oncological patient is the difficulty in managing chronic and neuropathic pain, which is frequently treated with opioid drugs for long periods of time. The different results in the evidence on safety of opioid use and cancer risk is a line of research that needs to be followed as it has a great impact in clinical practice, since even a small increase in risk would be relevant, given the high prevalence use of opioids (108).

#### β-Blockers and Nonsteroidal Anti-Inflammatory Drugs

The other options proposed could be the association of drugs with a β-adrenergic blocking effect to act on the catecholaminergic cascade of the stress response although the physiopathogenesis is not known yet (109), and selective COX-2 inhibitors that decrease the inflammatory response of prostaglandins, increased in the perioperative period (110). In clinical practice, the prescription of β-blockers and COX-2 inhibitors in a chronic way (β-blockers modulate the pharmacokinetics and pharmacodynamics of other anesthetic drugs, reducing intraoperative anesthetic requirements, as well as postoperative pain and nausea), has been shown to be a preventive intervention against the formation of primary tumors of various origins, including breast or colon, and has even managed to increase survival in patients who had already developed malignancies (14, 111). It is important to highlight the caution in prescribing cardioselective β-blockers in patients with a high risk of cardiac events. Recent prospective studies have introduced a noncardioselective β-blocker, such as propanolol, as it has demonstrated its safety (32, 112). An *in vivo* study demonstrated that administering a brief, clinically relevant dose of propanolol reduces tumor cell proliferation, lymphatic drainage, and metastatic colonization. In other studies, the perioperative administration of propanolol is associated with better survival compared to placebo in breast cancer (HR 0.50, 95% CI 0.32–0.80), especially at an early stage of the disease (HR 0.19, 95% CI: 0.06–0.60) (32). Evidence suggesting administration of β-blockers in the perioperative period has an increase in survival in patients with breast, lung, prostate, and ovarian cancer (106, 113, 114) are in controversy with a recent meta-analysis, where the administration of β-blockers had no effect on disease-free survival or overall survival in patients with cancer.

Nowadays, the potential effect of β-blockers has low-level evidence (112), and high-quality randomized controlled trials on the perioperative effect continue to be needed (115).

Regarding nonsteroidal anti-inflammatory drugs (NSAIDs), these are frequently used as pain relievers during the perioperative period. In surgical studies of breast and prostate cancer, short-term inhibition of COX-2 has shown an increase in apoptosis in neoplastic cells and a reduction in the proliferation, angiogenesis, and expression of HIF-1α. Furthermore, perioperative administration of NSAIDs in patients with breast or lung cancer improved short-term survival rates (14). A study analyzed 15,574 patients undergoing liver resection for HCC, and found a significant association between perioperative administration of NSAIDs and a 19% reduction in the rate of tumor recurrence in patients *vs*. placebo (HR 0, 81, 95% CI: 0.73–0.90) (32).

Therefore, it is suggested that pharmacological inhibition of both pathways could obtain synergistic effects and better results than acting individually. However, there are not too many studies evaluating the combination of effects (111). The combination of propanolol (40 mg daily) and etodolac (800 mg daily) 5 days prior to breast cancer surgery has been studied. The combined treatment compared to placebo achieved a lower increase in serum inflammatory markers (C reactive protein and IL-6) during surgery, and also reduced the expression of prometastatic transcription factors and the epithelial-mesenchymal transition. These findings demonstrate that a brief blockage of neuroinflammatory signaling during the perioperative period reduces the malignancy potential of neoplastic cells. But there are still many factors to study in this line, as well as defining the impact on long-term survival (32, 111).

Applied to clinical practice, the main proposal lies in the combined use of β-blockers and COX-2 inhibitors from the first few days before surgery to a few weeks after, since this measure could improve survival without involving large material or financial resources (116). Also, this pharmacological combination, as long as there are no major contraindications to its administration, has shown a good safety profile (14, 111).

## Conclusions

The perioperatory strategy is still under evaluation as a promising option to improve recurrence-free survival after cancer surgery with minimal cost.

Inhaled anesthetics and opioids have been associated with a higher rate of tumor recurrence and metastasis because they increase proinflammatory activity and decrease immune function. Reducing its use, or even replacing it as much as possible, could have a beneficial effect.

Propofol-based anesthesia and epidural anesthesia maintain the proper function of the immune system and reduce catecholaminergic and inflammatory responses. The studies *in vitro* and *in vivo* have shown to inhibit the proliferation and migration of cancer cells, induce apoptosis, and reduce metastatic development. Therefore, the protective effects against tumor spread mean that both propofol-based anesthesia and epidural anesthesia are proposed as possible ideal “immunoprotective” or “antitumor” anesthetic techniques in the management of patients with cancer.

Other possible pathways for blocking the catecholaminergic and inflammatory responses are based on the perioperatory administration of combined β-Blockers and COX-2 inhibitors, which until now have been shown to slow tumor development and even improve oncological prognosis.

Despite the growing number of studies addressing this topic, there are still many outstanding questions in the field of anesthesia and immunomodulation waiting to be answered by new prospective clinical trials. Thus, there appears to be a need to continue these avenues of research and obtain conclusive results to define a standardized and “antitumor” clinical practice in the perioperative management of the patient with cancer.

To regroup all the possible perioperatory actions discussed, they have been summarized in Table 3.

**Table 3:** Preclinical and clinical studies provide considerable evidence of how agents with antiadrenergic or anti-inflammatory properties, along with other specific anesthetic techniques, could have beneficial effects as possible perioperatory strategy.

**Perioperative intervention**		**Mechanism of action**	**Impact on the development of metastasis**	**Clinical evidence**
Actions that favor the Metastatic development	General anesthetics: • Inhaled agents	Increased levels of HIF-1α, VEGF, MMP, and TGF-β.	Increased migration and invasion of tumor cells.	Due to their negative impact on cancer prognosis, no specific clinical studies have been carried out, but rather these techniques have been used as a comparison against the possibilities that could be of benefit.
	•Intravenous agents • Opioid	Increased catecholamine synthesis.	Increased neuroendocrine stress response: – Immunosuppression – Increased angiogenesis.	
		Increased synthesis of proinflammatory mediators: PGE_2_.	Proinflammatory Microenvironment Formation (NPM): – Tumor proliferation – Metastatic progression	
		Decreased activity of lymphocytes, macrophages, and NK cells.	Immunosuppression.	
		Decreased activity of NK cells.	Immunosuppression.	
Decrease in the use of opioids	• Propofol	Anti-inflammatory effect, antioxidant.	Inhibition of the migration of tumor cells.	– Retrospective analysis of 7,030 patients compares survival in patients receiving anesthetic agents vs. propofol-based anesthesia (RH = 1.46 95% CI: 1.29–1.66) (103, 117). – Retrospective analysis associates propofol-based anesthesia in mastectomies with higher survival, compared to inhaled anesthesia (HR: 0.55, 95% CI: 0.31–0.97) (103, 117).
		Maintenance of NK cell function.	Immunoprotection.	
	Local anesthetics • Epidural anesthesia	Inhibition of the synthesis of catecholamines, proinflammatory mediators and cortisol.	Decreased neuroendocrine stress response: – Immunoprotection – Decreased angiogenesis – Decreased tumor spread	– Meta-analysis obtained positive association for neuroaxial anesthesia and survival improvement compared to general anesthesia (HR = 0.85, 95% CI: 0.741–0.981, *p* = 0.026) (14). – Prospective study of 42,000 patients older than 66 years with colorectal cancer. The use of AE is associated with a higher median survival (OR = 0.91 95% CI: 0.87–0.94, *p* < 0.001) (52).
Prevention	β-Blockers	B-adrenergic antagonism: inhibits the response to catecholamines (stress response).	– Decreased deleterious effect of catecholamines: Immunoprotection? – Reduction of tumor proliferation and colonization? – Nowadays, the potential effect of β-blockers has low-level evidence (112)	– Administration of β-blockers in the perioperative period as an increase in survival in patients with breast, lung, prostate and ovarian cancer (106, 113, 114) are in controversy with a recent meta-analysis: administration of β-blockers had no effect on disease-free survival or overall survival in patients with cancer.
	NSAIDs • COX-2 inhibitor • COX-2 inhibitorsand β-Blockers	Reduction in PGE_2_ levels. VEGF inhibition. NK cell activity maintenance	– Inhibits the formation of the proinflammatory microenvironment. – Angiogenesis reduction. – Immunoprotection	– Retrospective study: 15,574 patients undergoing liver resection. Administering perioperative NSAIDs reduces tumor recurrence and increases survival (HR = 0.81, 95% CI: 0.73–0.90) (103, 117). – The use of perioperative NSAIDs is associated with prognostic improvement in breast and colorectal cancer (103, 117). – Combination of propanolol (40 mg daily) and etodolac (800 mg daily) 5 days prior to breast cancer surgery during the perioperative period reduces neoplasic cells malignancy potential (111).
		Lower increase in serum inflammatory markers. Reduced expression of prometastatic transcription factors and epithelial-mesenchymal transition.	Blockage of neuroinflammatory signaling.	

## Summary

Cancer is one of the main causes of morbidity and mortality in the world. Surgery is the “gold standard” strategy used in the therapeutic management of cancer. In recent years, many studies have investigated the link between the development of metastases and immunosuppression related to surgery and anesthesia. Anesthetic drugs can induce essential pathophysiological changes in metastatic development in relation to proliferation, angiogenesis, and cellular apoptosis. This review aims to summarize the evidence on anesthetic agents and techniques used during oncological surgery, which promote metastatic development, and if there are “antitumor” techniques that increase the survival of the oncological patient.

## Author Contributions

SS, AL, and ML: conceptualization and writing-review and editing. SS, CM, and AL: methodology. SS, CM, and ML: investigation. SS and AL: writing—original draft preparation. CM: visualization. SS and ML: supervision. AL and CM: project administration. All authors have read and agreed to the published version of the manuscript.

## Conflict of Interest

The authors declare that the research was conducted in the absence of any commercial or financial relationships that could be construed as a potential conflict of interest.

## Publisher's Note

All claims expressed in this article are solely those of the authors and do not necessarily represent those of their affiliated organizations, or those of the publisher, the editors and the reviewers. Any product that may be evaluated in this article, or claim that may be made by its manufacturer, is not guaranteed or endorsed by the publisher.
